# Low‐cost Kinect Version 2 imaging system for breath hold monitoring and gating: Proof of concept study for breast cancer VMAT radiotherapy

**DOI:** 10.1002/acm2.12286

**Published:** 2018-03-13

**Authors:** David M. Edmunds, Lone Gothard, Komel Khabra, Anna Kirby, Poonam Madhale, Helen McNair, David Roberts, KK Tang, Richard Symonds‐Tayler, Fatemeh Tahavori, Kevin Wells, Ellen Donovan

**Affiliations:** ^1^ Department of Physics The Royal Marsden NHS Foundation Trust London UK; ^2^ The Institute of Cancer Research London UK; ^3^ Department of Physics University of Surrey Guildford UK; ^4^ Centre for Vision, Speech and Signal Processing University of Surrey Guildford UK

**Keywords:** radiotherapy, motion monitoring, sensors, respiratory gating

## Abstract

Voluntary inspiration breath hold (VIBH) for left breast cancer patients has been shown to be a safe and effective method of reducing radiation dose to the heart. Currently, VIBH protocol compliance is monitored visually. In this work, we establish whether it is possible to gate the delivery of radiation from an Elekta linac using the Microsoft Kinect version 2 (Kinect v2) depth sensor to measure a patient breathing signal. This would allow contactless monitoring during VMAT treatment, as an alternative to equipment–assisted methods such as active breathing control (ABC). Breathing traces were acquired from six left breast radiotherapy patients during VIBH. We developed a gating interface to an Elekta linac, using the depth signal from a Kinect v2 to control radiation delivery to a programmable motion platform following patient breathing patterns. Radiation dose to a moving phantom with gating was verified using point dose measurements and a Delta4 verification phantom. 60 breathing traces were obtained with an acquisition success rate of 100%. Point dose measurements for gated deliveries to a moving phantom agreed to within 0.5% of ungated delivery to a static phantom using both a conventional and VMAT treatment plan. Dose measurements with the verification phantom showed that there was a median dose difference of better than 0.5% and a mean (3% 3 mm) gamma index of 92.6% for gated deliveries when using static phantom data as a reference. It is possible to use a Kinect v2 device to monitor voluntary breath hold protocol compliance in a cohort of left breast radiotherapy patients. Furthermore, it is possible to use the signal from a Kinect v2 to gate an Elekta linac to deliver radiation only during the peak inhale VIBH phase.

## 1 INTRODUCTION

Breast cancer is the most common malignancy in women in the UK, with more than 40,000 new cases diagnosed each year.^1^ Surgery followed by radiotherapy improves local control and survival such that rates of local tumor relapse in the breast are now approximately 3% at 5 yr.^2^, ^3^ However, breast radiotherapy is also associated with a 1% increase in nonbreast‐cancer‐related deaths at 15 yr, 90% of which are cardiovascular in origin.^4^, ^5^ Given the increasing incidence of breast cancer, and the large numbers of survivors in the population, it is imperative that the improvements in breast cancer mortality are not compromised by nonbreast‐cancer deaths. This may be achieved by reducing the radiation dose to the heart from radiotherapy. One approach to achieving this is deep inspiration breath‐hold, using either equipment‐assisted or voluntary inspiration breath‐hold (VIBH) techniques.^6^, ^7^

VIBH techniques are straightforward and cost‐effective to implement, as they use equipment already available in the radiotherapy treatment room. This includes the field light indicators and lasers used to aid patient setup. The field lights or lasers plus skin reference marks are viewed via CCTV cameras in the control areas and provide confidence that the patient is holding her/his breath in a reproducible way during radiotherapy delivery.

More complex approaches to delivering radiotherapy such as Volumetric Modulated Arc Therapy (VMAT) are likely to become standard treatments for selected groups of patients.^8^ There is potential for the light fields/lasers to be occluded from view by the gantry if used for these treatments, which may inhibit visual monitoring of VIBH, hence requiring equipment‐assisted methods to be used. In addition, the visual monitoring method, whilst shown to be safe and effective,^9^ does not allow for automatic gating of the linac in the event of a sudden patient movement such as a cough. We propose using the Microsoft Kinect version 2 (Kinect v2) device as a simple, low cost, noncontact monitoring method as a potential solution.

The Kinect v2 was originally designed as a motion‐tracking peripheral for the Microsoft Xbox One games console. The sensor contains a standard high definition (HD) camcorder, an infrared transmitter and receiver and an array of microphones for positional sound detection. An infrared time‐of‐flight (TOF) technique is used to estimate the distance from the camera to objects in the room. The Kinect v2 has significantly better performance characteristics than the older Kinect v1 sensor that has been in the literature for several years, including a higher resolution depth sensor (512 × 424 vs. 320 × 240), higher resolution color sensor (1920 × 1080 vs. 640 × 480) and wider field of view (70° H, 60° V vs. 57° H 43° V).

The Kinect v2 has already been used for external head motion tracking in brain PET scans,^10^ respiratory motion correction in PET scans^11^ and respiratory motion tracking in radiotherapy using a marker‐based system.^12^ Previous work has shown that the sensor has distance accuracy and precision of 1 to 2 mm after calibration, which is sufficient for breath‐hold monitoring where changes are 5 to 10 mm in magnitude.^10^, ^11^, ^13^, ^14^, ^15^ In our previous work,^13^ we demonstrated that the Kinect v2 can be used safely in a radiation environment without image distortions caused by the radiation beam. The work reported here can be divided into two components:

1. We performed the first clinical study using the Kinect v2 in breast radiotherapy, with markerless tracking. This was a clinical feasibility study of observational design. Six breast cancer radiotherapy patients were monitored in a standard supine treatment position while performing a VIBH breathing protocol. Distance information was extracted using the Kinect v2, and used to obtain breathing traces from the patients.
2. We developed a gating interface to an Elekta linac, using the depth signal from a Kinect v2 to control the delivery of radiation to a programmable motion platform. Patient breathing traces obtained from part 1) were used as movement patterns for the motion platform, to verify that radiation was correctly delivered while the gating system was in use.

One aim of this work is to demonstrate that it is possible to monitor compliance with the VIBH protocol and acquire breathing traces from patients using the Kinect v2. The second aim is to show that it is possible to gate radiation delivery based on a signal from a Kinect v2, derived from patient motion patterns applied to a programmable motion platform.

## 2 MATERIALS AND METHODS

### 2.A Clinical study protocol and recruitment

The clinical study design was observational, noninterventional, and nonrandomized. The study was designed to test the hypothesis that repeated VIBHs can be monitored with a non contact device (Kinect v2 system) in a cohort of patients in the supine treatment position on an angled breast board, on a radiotherapy treatment unit during the procedure for both standard two field whole breast and VMAT radiotherapy.

**Figure 1:**
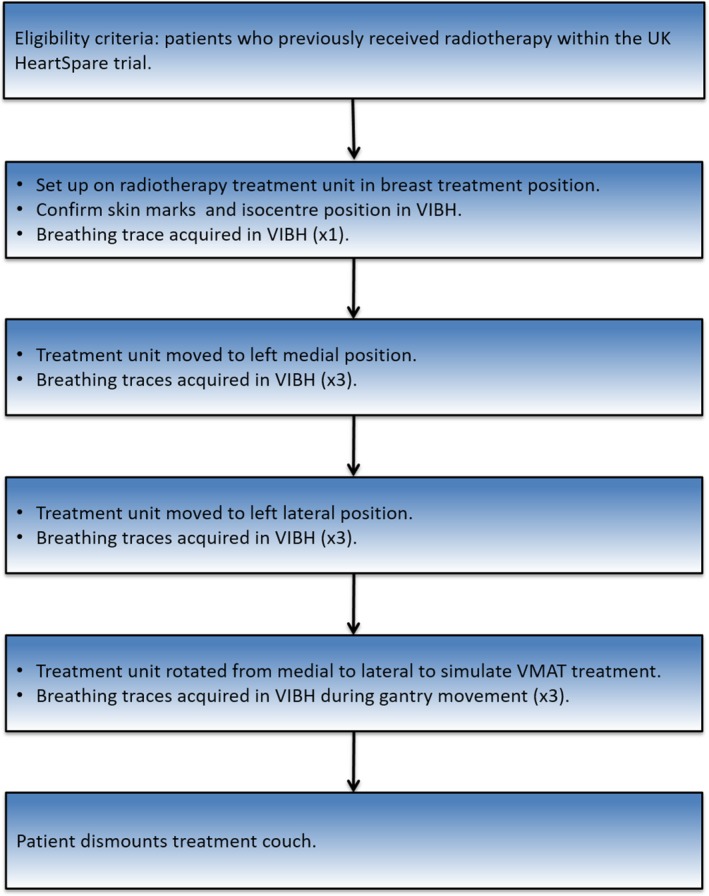
Workflow diagram for clinical study.

The primary end point was the percentage of planned breath holds for which complete monitoring traces were acquired using Kinect v2. A complete trace was defined by a sequence of free breathing, followed by 20 s of breath hold, followed by free breathing. The secondary end point was the percentage of planned breath holds for which complete traces were acquired at each of the defined time points of the setup and treatment phases over the patient cohort. The purpose of this end point was to identify the time point(s) in the process where failure of data collection occurred. Figure 1 shows a diagram of the patient pathway used in this study.

46 monitored breath‐holds from five patients were required for 90% power for an expected 99% success rate, ruling out any rate <90% at 5% significance level. The final required sample size of 50 breath holds allowed for any patients who were not willing/able to hold their breath at any of the time points in the process. The monitoring method was considered feasible if there were no more than one unsuccessful collection of a complete trace. Patients who had previously been treated with left breast radiotherapy using a VIBH breathing protocol as participants in the UK HeartSpare trial were eligible for this feasibility study.^6^, ^9^ Local and external ethics approval was obtained from the Leicester South NRES East Midlands Research Ethics Committee and 10 patients were invited to participate.

### 2.B Clinical study experimental setup

**Figure 2:**
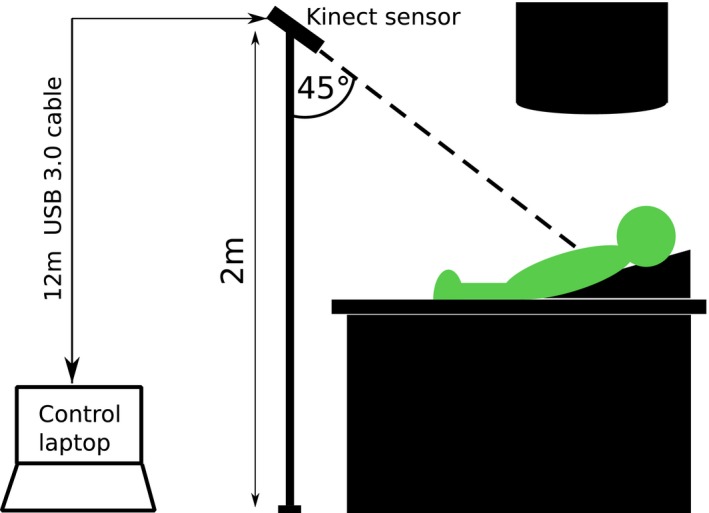
Diagram of clinical study experimental setup. A Kinect v2 sensor was connected to a dedicated control laptop in the treatment room control area using a 12 m USB 3.0 active repeater cable.

A Kinect v2 was setup on a tripod system in an Elekta treatment bunker as shown in Fig. 2. In previous work, a calibration procedure for the Kinect v2 was established, and the temperature stability of the device was investigated.^13^ The clinical study protocol was then followed according to Fig. 1, and breath hold data were acquired using the Kinect v2 sensor. The light field was also used to monitor breath holds visually, as per clinical practice.

**Figure 3:**
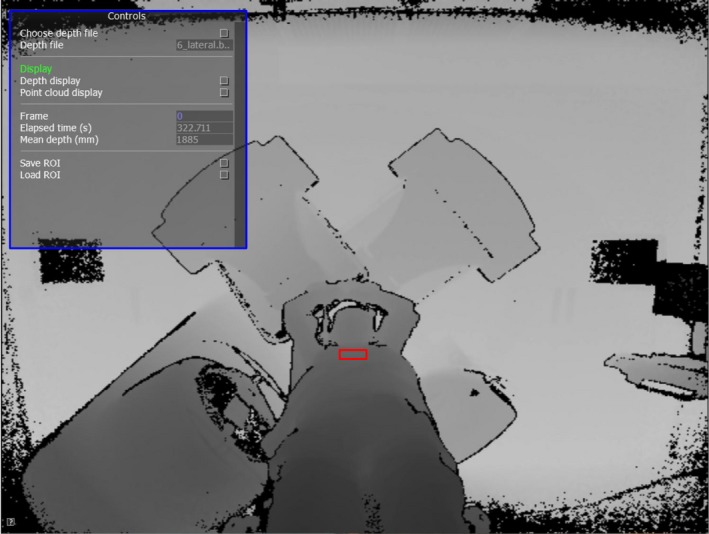
Screenshot of custom C++ software used to record Kinect depth data. Software controls for manipulating depth frame data are shown in the top left (blue rectangle). A patient is visible on the couch, supported by a breast board. The entire depth frame from the sensor is recorded at a resolution of 512 × 424 pixels and frame rate of 30 fps into a lossless binary file for later analysis. The user selects a ROI (red rectangle) on the patient's upper sternum region, with an area of approximately 300 pixels. The mean distance from the Kinect v2 to this ROI is then calculated as a function of time to form a breathing trace signal.

To acquire breath hold data from the Kinect v2, custom C++ software was written using the free Kinect for Windows Software Development Kit (SDK) 2.0 (see Fig. 3). Patient volunteers were setup on the couch with a breast board, according to the parameters in their original treatment plans. Two experienced radiographers performed the patient setup procedure. Patients were setup to their original treatment tattoos, with their peak inhale voluntary breath‐hold position marked on the skin.

### 2.C. Gating study

**Figure 4:**
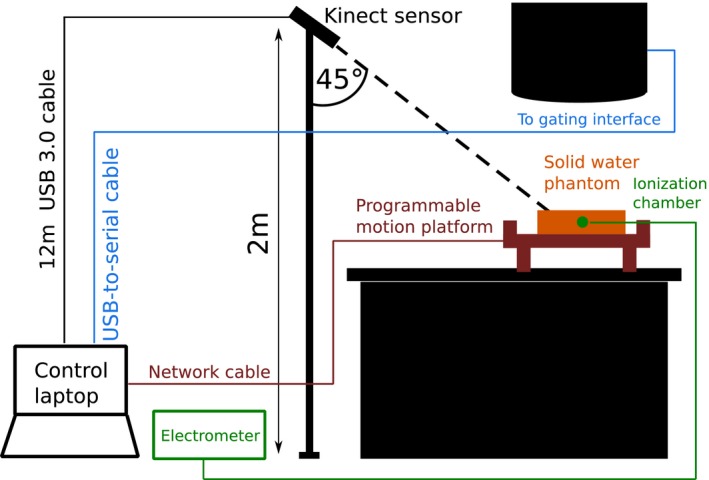
Experimental apparatus for gating experiment. A Kinect v2 sensor was setup as in Fig. 2. An in‐house, high‐precision programmable motion platform was placed on the treatment couch. A solid water phantom was positioned on the motion platform, with a NE2571 Farmer chamber inserted inside. A NE2560 electrometer was used in conjunction with the Farmer chamber to make point dose measurements. The control laptop was connected directly to the linac gating interface using a USB‐to‐serial connection. For the Delta4 experiments, the solid water phantom was replaced by a Delta4 phantom connected to a control PC via an Ethernet connection.

A separate set of experiments was carried out to investigate whether the depth signal from a Kinect v2 system could be used to gate an Elekta linac via the previously tested Elekta Response ™ gating interface.^16^, ^17^ See Fig. 4 for a diagram of the experimental setup.

Patient breathing traces acquired as described in Section 2.B were used as input to the motion platform. In order to avoid exceeding the physical constraints of the motion platform, some modifications to the patient breathing traces were required:

1. A Savitzky‐Golay smoothing filter was applied to avoid velocities greater than the motion platform's limit of 3 cm/s.
2. The Kinect v2 measured a 1D signal *d*_Kinect_, the scalar distance between the patient's chest and the sensor. In order to accurately reproduce this signal with the motion platform, it had to be resolved into a 2D signal to move the platform along its x and z axes simultaneously. Since the Kinect v2 was angled at 45° from horizontal during recording, it sufficed to use $x,z=d_{\mathrm{Kinect}}/\sqrt{2}$.
3. All amplitudes were reduced by 20% to avoid exceeding the platform's maximum z axis motion range of 5 cm.

Three situations were investigated:

1. A 200 MU 10 × 10 cm^2^ single beam delivery for 0.3 min with the gantry fixed at 0°(ionization chamber point dose in a solid water phantom).
2. A 250 MU 20 × 20 cm^2^ simple conformal arc delivery for 0.9 min with the gantry rotating counter‐ clockwise between 140° and −50° at constant dose rate (ionization chamber point dose in a solid water phantom).
3. A clinical VMAT plan to treat whole breast and superclavicular nodes, with a single clockwise arc from 333 to 179 with 484 MU per fraction. Beam‐on time was 1.7 min. The solid water phantom was replaced by a Delta4 4D verification phantom [Scandidos, Uppsala, Sweden]. This was to enable a spatial comparison of dose between ungated delivery to a static phantom, and gated deliveries using the 2D dose maps provided by the phantom.

For each modified breathing trace:

1. The motion platform was allowed to move until it reached the peak inhale position of the first breath hold, and was then held fixed.
2. Couch adjustments were used to move the solid water phantom surface to 100 cm SSD with the ionization chamber at 5 cm deep/Delta4 phantom to the clinical plan isocenter.
3. Radiation was delivered with the motion platform in this fixed reference position, and accumulated charge was recorded using the electrometer to obtain a reference point dose measurement. This was repeated three times. (Delta4 reference was obtained for the clinical VMAT delivery)
4. Our in‐house software was used to select a rectangular region of interest (ROI) with an area of 300 pixels on the surface of the solid water/Delta4 phantom to obtain *d*_Kinect_ at peak inhale. This was used to manually select a gating window of *d*_Kinect_ ± *τ* mm using our software. We chose a maximum allowable value of *τ *= 5 mm to prevent overlap between free breathing and breath hold signals. When the motion platform moved outside of this gating window, an inhibit signal was sent by our software to the linac gating interface over a USB‐to‐serial connection, preventing radiation delivery.
5. The motion platform was then reset and allowed to move freely following the input modified breathing trace. Radiation was delivered with gating active. Accumulated charge was recorded using the electrometer. This was repeated three times.

For each case, the breathing trace acquired from the patients during simulated VMAT treatment was used for dose verification purposes. Gamma analysis parameters produced by the Delta4 software were used to compare ungated with gated doses.

## 3 RESULTS

### 3.A Clinical study

10 patients were contacted and six responded favorably and participated in the study. All patient data were collected in a single day. Both the primary and the secondary end points of the clinical study were met as traces at all planned time points were successfully acquired with the Kinect v2, giving a total of 60 breath hold traces.

**Figure 5:**
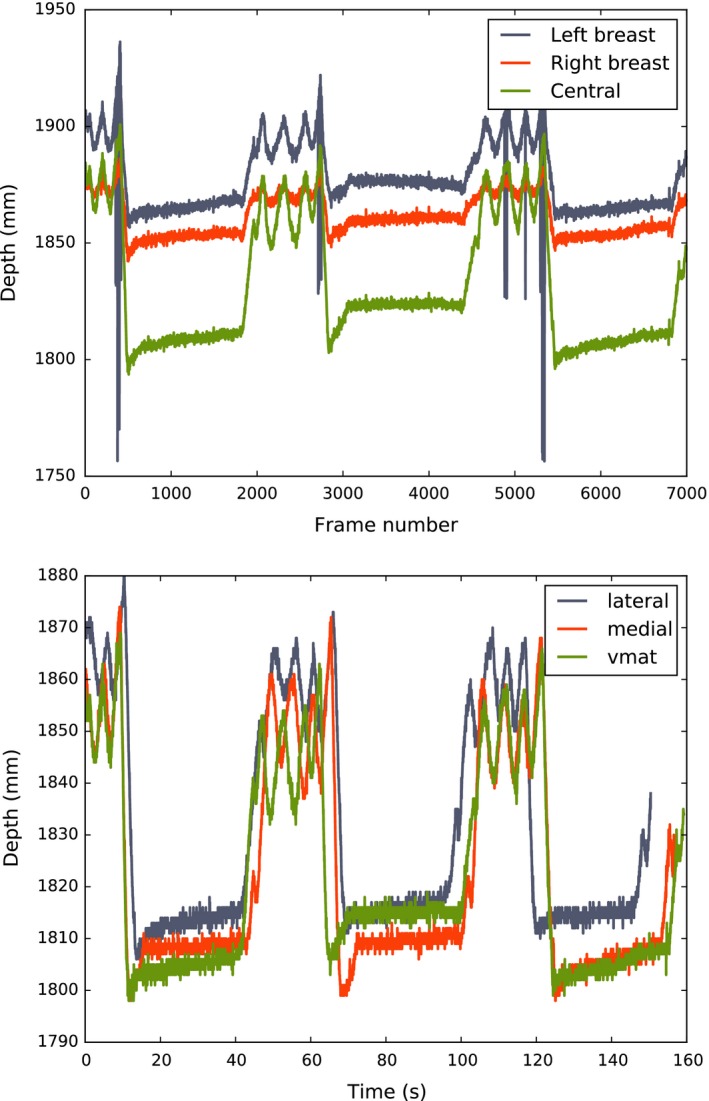
Top: Example breathing traces extracted from VMAT data for patient 6, using three different ROIs, drawn on the left breast, right breast and central chest region between the breasts respectively. A 5 × 5 square selection of pixels was used for all three ROIs. This figure demonstrates the sensitivity of the extracted breathing trace to the exact position of the selected ROI. Bottom: Comparison of breathing traces extracted from breath hold data for patient 6 using a central ROI. The breath hold data was recorded with the gantry in the lateral and medial treatment positions, and while the gantry was rotating to simulate a VMAT treatment. All breathing traces appear inverted, because d_Kinect_ decreases as the patient inhales and moves closer to the sensor.

Figure 5 shows the breathing traces extracted from the Kinect v2 depth data acquired for a single patient, using three different ROI selections. It can be seen that the traces are sensitive to the exact position of the ROI used. Figure 5 also shows all three breathing traces extracted for patient 6 for the three different gantry positions recorded. Empirically, the “central” ROI was found to be the most consistent and easy to locate in the patient depth images, so this was used for each patient in all further analysis.

### 3.B Gating study

**Figure 6:**
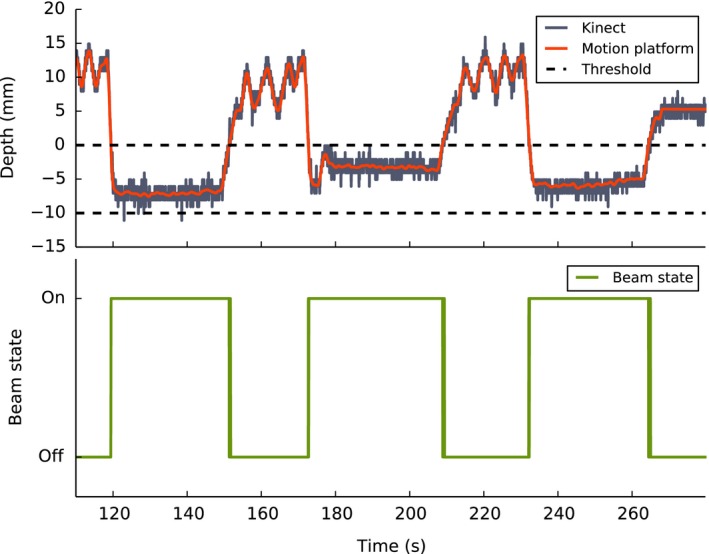
Example of Kinect v2‐monitored radiation delivery during breath hold to a motion platform with gating active. Corresponding beam state signal is also shown.

See Fig. 6 for an example of Kinect v2‐monitored, gated radiation delivery to the solid water phantom. The Kinect v2 was successfully able to track the motion platform, and gating signals were sent at the correct times. See Table 1 for a comparison of accumulated charge between gated and ungated deliveries. All gated deliveries agreed with the reference data to within 0.5%, suggesting that radiation was delivered correctly, when the motion phantom was within the preselected gating window only. Traces from patients 5 and 6 were excluded because it was not possible to complete a successful radiation delivery with a threshold value *τ *< 5 mm without the linac terminating due to an extended period of beam inactivity.

**Table 1:** Top: Comparison of gated vs. ungated charge recorded by electrometer for a 200 MU static beam delivery. Bottom: Comparison of gated vs. ungated charge recorded by electrometer for a 250 MU simple conformal arc with a 20 × 20 cm^2^ field size

Trace	Ungated charge (nC)	With gating (nC)	Ratio (gated/ungated)
1	37.534	37.340	0.995
2	37.508	37.523	1.000
3	37.689	37.745	1.002
4	37.636	37.614	0.999

1	45.248	45.161	0.998
2	45.195	45.164	0.999
3	45.312	45.219	0.998
4	45.229	45.164	0.999

Table 2 shows a comparison of dose distributions between gated and ungated radiation deliveries as measured by the Delta4 system. In all cases, dose distributions from the static phantom with ungated delivery were used as a reference point in the Delta4 software, and the gated dose distributions were compared with these references. Median dose differences were better than 0.5% in all cases, and the mean (3% 3 mm) gamma index was 92.6%.

**Table 2:** Measured dose data from the Delta4 system. Median dose difference and gamma index (3% 3 mm) are shown. In each case, the ungated radiation deliveries were used as the reference data and compared to the gated radiation deliveries, so the percentages in this table represent percentage agreement between gated and ungated deliveries. Breathing traces for each patient were used. One whole breast radiotherapy (WBRT) standard two‐field plan is included for comparision with the more complex VMAT plans. The “free breathing” trace was a sinusoid with an amplitude of 10 mm and period of 1 s. The gamma index result for patient 6 was poor because this patient had a particularly noisy breathing trace, which caused the breathing signal to jump in and out of the gating threshold rapidly

Plan type	Case	Median dose difference (%)	Gamma index (%)
VMAT	1	−0.1	98.0
VMAT	2	0.2	100.0
VMAT	3	0.5	80.8
VMAT	4	0.0	98.7
VMAT	5	0.0	95.6
VMAT	6	−0.1	68.4
WBRT	1	−0.1	99.0
“Free breathing”		0.2	100.0

## 4 DISCUSSION

As previously reported,^13^ the Kinect v2 is able to track motion patterns with a root mean squared accuracy of approximately 1.5 mm. Hardware latency causes a delay between a beam on signal and the commencement of radiation delivery, and vice versa for beam off. Previous studies have found beam‐on delays ranging between 220 ms^18^ and nearly 1 s.^17^ This raises questions about the dosimetric consequences of gating an Elekta linac during free breathing. Fortunately, for our application, where radiation is delivered only during breath holds that last approximately 20 s, the impact of this latency is negligible. This is confirmed by our point dose measurements, which show agreement between gated and nongated deliveries of better than 99.5%.

The Delta4 dose distribution data showed a range of agreements between the gated and ungated radiation deliveries, with the majority being clinically acceptable. This work was a proof of principle, and an in–depth investigation of the reasons for the variations would be required if this approach was to be considered for patient treatment.

In this work, we used a rather large total gating window width of ±0.5 cm. In the course of our experiments, we discovered that *d*_Kinect_ to a completely stationary target could vary by as much as 7 mm as the gantry was rotated during a simulated VMAT delivery. This is probably due to the infrared scatter from the linac head as it rotates into the sensor's field of view. With an angular‐dependent calibration, it would be possible to correct the sensor's output to take account of this effect. In turn, it would be possible to reduce the size of the gating window used. A gating window of ±2 mm would be realistic. Colgan et al. showed that measurable movement from movie loops recorded during treatment in breath hold did not exceed 3mm and the median displacement was 1.5 mm.^9^

It is still common not to define a PTV margin explicitly in standard whole breast radiotherapy although where this is done margins are typically 10 mm; for complex treatments (e.g., VMAT for breast and involved nodes) they may be reduced to 5 mm. A gating window of ±2 mm is reasonable in this context.

In Fig. 5, it can be seen that there is some lack of consistency for breathing traces recorded for patient 6 during three different simulated treatments. Either this is a result of natural variations in the patient's breathing pattern, or the effect of fatigue following multiple repeated breath holds in a short time period. This is the subject of further investigation.

We found that *d*_Kinect_ is very sensitive to the position of the selected ROI on the patient's chest. Selecting the ROI poorly leads to large fluctuations in the measured breath hold signal, which makes selecting an appropriate gating window challenging. We found that the optimal ROI position for a stable signal was centrally on the upper torso, just below the breasts. Currently, this ROI has to be identified and drawn manually by the software operator. A method to define this ROI automatically is a subject of further research.

The position of the Kinect v2 sensor in the treatment room also requires optimization. We positioned the sensor on a tripod at the end of the couch, but a clearer view of the patient's chest region may be obtained by mounting the sensor on the ceiling, directly in front of the gantry above the couch. This may also assist with optimal ROI selection.

In this work, gating signals were triggered using the raw, unfiltered and unsmoothed breathing trace data. It is possible that noise in the breathing traces can cause the trace signal to momentarily enter the gating window and trigger an instantaneous gate on/off signal at an undesirable point in the breathing cycle. This issue can be avoided by applying a smoothing filter to the incoming breathing signal, or building in a hysteresis function which will only trigger gating if the signal remains inside the gating window for a predefined amount of time.

There is already a wide variety of commercial optical and infrared surface imaging systems available for radiotherapy. These include VisionRT,^19^ NDI Polaris^20^ and C‐RAD Catalyst.^21^ These systems have benefits both for breath hold control and for patient setup. For example, they can be used for checking and correcting the relative positions of the breast and thorax in larger breasted patients.

The main advantage of a commodity depth sensor such as the Kinect v2 is one of cost, with a sensor, control laptop and all other hardware required available for around $1,400. The equivalent commercial systems can cost upwards of $250,000. Of course, the Kinect v2 has not gone through the required regulatory process to be certified as a medical device, which may complicate its widespread deployment in a clinical environment. However, it provides a very cost‐effective alternative to the commercial systems for the purposes of research and development. It could also be used as a quality assurance tool for gated techniques which are triggered by other devices. The low cost of the Kinect sensor also makes it feasible to have multiple units installed in the treatment room, which may allow full 3D reconstructions of the patient's body surface contour in the future.

## 5 CONCLUSION

We have performed a proof–of–concept study using the Kinect v2 for patient monitoring in radiotherapy, and developed a gating interface using the Kinect v2 to control radiation delivery with an Elekta linac. It is possible to use a Kinect v2 device to monitor voluntary breath hold protocol compliance in a cohort of left breast radiotherapy patients. Furthermore, it is possible to use the signal from a Kinect v2 to gate an Elekta linac to deliver radiation only during the peak inhale VIBH phase.

## ACKNOWLEDGMENTS

We thank Cheryl Taylor for her assistance with the clinical study, and we are very grateful to all the patients who volunteered to take part. The work was undertaken in The Royal Marsden NHS Foundation Trust which receives a proportion of its funding from the NHS Executive; the views expressed in this publication are those of the authors and not necessarily those of the NHS executive. We acknowledge NHS funding to the NIHR Biomedical Research Centre and the support of the NIHR, through the South London Cancer Research Network. This report is independent research arising from a Career Development Fellowship award, Ellen Donovan, CDF‐2013‐06‐005 supported by the National Institute for Health Research. The views expressed in this publication are those of the author(s) and not necessarily those of the NHS, the National Institute for Health Research, or the Department of Health.

## CONFLICT OF INTEREST

The authors have no relevant conflicts of interest to disclose.
